# Rapid identification of human muscle disease with fibre optic Raman spectroscopy†

**DOI:** 10.1039/d1an01932e

**Published:** 2022-04-21

**Authors:** James J. P. Alix, Maria Plesia, Gavin R. Lloyd, Alexander P. Dudgeon, Catherine A. Kendall, Channa Hewamadduma, Marios Hadjivassiliou, Christopher J. McDermott, Gráinne S. Gorman, Robert W. Taylor, Pamela J. Shaw, John C. C. Day

**Affiliations:** Sheffield Institute for Translational Neuroscience, University of Sheffield UK j.alix@sheffield.ac.uk; Neuroscience Institute, University of Sheffield UK; Phenome Centre Birmingham, University of Birmingham UK; Biophotonics Research Unit, Gloucestershire Hospitals NHS Foundation Trust UK; Biomedical Spectroscopy, School of Physics and Astronomy, University of Exeter UK; Department of Neurology, Sheffield Teaching Hospitals NHS Foundation Trust UK; Wellcome Centre for Mitochondrial Research, Translational and Clinical Research Institute, Faculty of Medical Sciences, Newcastle University Newcastle upon Tyne UK; NHS Highly Specialised Service for Rare Mitochondrial Disorders, Newcastle upon Tyne Hospitals NHS Foundation Trust Newcastle upon Tyne UK; Interface Analysis Centre, School of Physics, University of Bristol UK

## Abstract

The diagnosis of muscle disorders (“myopathies”) can be challenging and new biomarkers of disease are required to enhance clinical practice and research. Despite advances in areas such as imaging and genomic medicine, muscle biopsy remains an important but time-consuming investigation. Raman spectroscopy is a vibrational spectroscopy application that could provide a rapid analysis of muscle tissue, as it requires no sample preparation and is simple to perform. Here, we investigated the feasibility of using a miniaturised, portable fibre optic Raman system for the rapid identification of muscle disease. Samples were assessed from 27 patients with a final clinico-pathological diagnosis of a myopathy and 17 patients in whom investigations and clinical follow-up excluded myopathy. Multivariate classification techniques achieved accuracies ranging between 71–77%. To explore the potential of Raman spectroscopy to identify different myopathies, patients were subdivided into mitochondrial and non-mitochondrial myopathy groups. Classification accuracies were between 74–89%. Observed spectral changes were related to changes in protein structure. These data indicate fibre optic Raman spectroscopy is a promising technique for the rapid identification of muscle disease that could provide real time diagnostic information. The application of fibre optic Raman technology raises the prospect of *in vivo* bedside testing for muscle diseases which would significantly streamline the diagnostic pathway of these disorders.

## Abstract 2

We demonstrate the use of fibre optic Raman spectroscopy for the rapid identification of muscle disorders.

## Graphical abstract

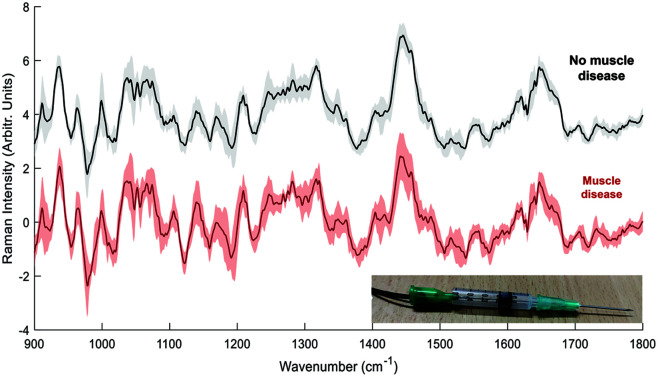

## Introduction

Muscle diseases, or myopathies, are a heterogeneous group of conditions that can be characterised by histological and functional abnormalities of skeletal muscle. Myopathies can present at any time of life and can cause a number of symptoms such as weakness, muscle pain and fatigue, depending upon the underlying cause. These symptoms typically affect proximal muscles in the upper and lower limbs, particularly the shoulder and pelvic-girdle muscle groups, respectively.^1^

The classification of muscle disease is complex. At the most basic level, muscle disorders can be described as hereditary (*e.g.* Duchenne muscular dystrophy), or acquired (*e.g.* inflammatory) but extensive subdivisions exist within each.^2–4^ The wide range of symptoms and their overlap across different aetiologies can provide a diagnostic challenge. As a result, even in healthcare systems in which a neurologist can be the first point of contact, the average time to diagnosis is >3 years.^5^ The chronicity of most muscle diseases results in substantial morbidity and a high socioeconomic cost.^6,7^ The development of rapid, simple tests for both clinical practice and research remains an area of unmet need.

At present, the diagnostic challenge posed by myopathies is met through clinical assessment followed by a composite of investigations, all with their own advantages/disadvantages. For example, simple blood tests can include non-specific tests, such as creatinine kinase, a muscle enzyme that can have some value in monitoring disease activity in certain conditions^8^ but is poorly predictive of underlying muscle disease.^9^ Nerve conduction studies and electromyography (EMG) involve recording the electrical activity of nerves and muscles, respectively. These tests are useful in defining any concomitant nerve and/or neuromuscular junction involvement and the topographical distribution of muscle abnormalities, all of which can give clues as to the underlying aetiology.^10^ However, the findings are ultimately non-specific. Imaging techniques such as magnetic resonance imaging (MRI) or ultrasound (USS) can also build up a picture of the distribution of abnormal muscles.^11^ Both EMG and imaging can provide information on which muscle to target for biopsy. Integration of next-generation sequencing strategies, such as whole exome or whole-genome sequencing, into diagnostic pathways is driving a ‘genomics first’ approach in the diagnosis of some myopathies, such as congenital and mitochondrial myopathies.^12,13^

Despite the wide range of investigations available, muscle biopsy remains a key component of the diagnostic process, allowing for genetic, histological and biochemical assays on the affected tissue. For example, in mitochondrial disease, some disorders of mitochondrial DNA maintenance only manifest pathology in post-mitotic muscle. In addition, there are some pathological genotypes that can only be detected in this tissue *e.g.* muscle restricted mitochondrial DNA variants. Thus, a muscle biopsy can provide a clinico-pathological diagnosis and/or guide genetic testing and counselling. Furthermore, muscle biopsies can also be used to provide functional correlates for genetic variants of uncertain significance, facilitating their clinical interpretation.^14,15^ A wide range of abnormalities can be seen on biopsy and as a result the processing of these samples is complex, requiring highly specialised, time-consuming techniques and expert analysis.

Raman spectroscopy uses monochromatic light to probe the chemical composition of a sample. By analysing the frequency content of inelastically scattered light, information on molecular bonds is gained. In tissue analyses this achieves a “biochemical fingerprint” of the sample.^16^ Spontaneous Raman spectroscopy is the simplest Raman application to implement, requiring only a single laser and no sample preparation. Rapid improvement in the technical performance of fibre optic formats offers the potential of real time tissue analysis in a range of clinical settings.^17^ Recently, Raman spectroscopy has shown promise in the identification of neurological disorders,^18,19^ including myopathies.^20–22^

In the present study we have used *ex vivo* human muscle samples from patients with either genetically confirmed muscle disease, or patients under investigation for suspected muscle disease. Our aim was to test the hypothesis that portable fibre optic Raman spectroscopy can identify abnormal muscle without any sample preparation.

## Methods

### Participants

A total of 44 participants were included with one muscle sample per participant. Samples were collected through either an open biopsy or conchotome needle biopsy and snap frozen in either Sheffield or Newcastle, in accordance with local standard operating procedures. Patients were recruited and samples collected either at the time of investigation for a possible myopathy, or following genetic confirmation of mitochondrial myopathy. Patients undergoing investigation for myopathy were followed up until investigations revealed a final diagnosis. For data analysis, samples were initially divided into two groups: patients in whom the muscle biopsy and clinical context arrived at a diagnosis of “muscle disease” and patients with alternative diagnoses (“no muscle disease”). The “muscle disease” group was then subdivided into patients with a genetic diagnosis of mitochondrial disease, hereafter referred to as “mitochondrial myopathy”, and those with a “non-mitochondrial myopathy”. The use of human tissue was approved by NHS Research Ethics committees (references 16/YH/0261 and 09/H0906/75); good clinical practice (GCP) guidelines were followed and participants provided appropriate informed consent. The basic demographic and clinical characteristics of the patients are given in Table 1. The muscle biopsy findings and further clinical details are presented in ESI Table 1.†

**Table 1:** Clinical details

	Mitochondrial myopathy (*n* = 14)	Non-mitochondrial myopathy (*n* = 13)	No muscle disease (*n* = 17)	*P* value (statistical test)
**Gender**				
Male : female	8 : 6	9 : 4	6 : 11	0.17 (*X*^2^)
**Age**				
Mean (range, years)	51 (29–80)	52 (22–73)	55 (23–80)	0.7 (ANOVA)
**Muscle biopsied**				
Biceps	—	2	4	
Quadriceps	3	6	5	
Tibialis Anterior	11	—	—	
Deltoid	1	6	9	
**Clinico-pathological diagnoses (*n*)**				
m.3243A > G variant	11			
POLG-related mitochondrial disease	3			
Single large-scale mtDNA deletion	1			
Metabolic myopathy		1		
Myopathy: unknown aetiology		5		
Dystrophic myopathy		4		
Inclusion body myositis		1		
Subacute idiopathic inflammatory myopathy		2		
Vascular dementia			1	
Cerebellar ataxia			8	
Lumbar radiculopathy			1	
Myaesthenia gravis			1	
Fibromyalgia			1	
Elevated CK: malignant hyperthermia			1	
Elevated CK: statin-related			2	
Diabatic neuropathy			1	
Medical myelopathy			1	
Sensory ganglionopathy			1	

Mitochondria are complex organelles under the genetic control of both the nuclear and mitochondrial genomes and both genomes are associated with mitochondrial myopathy. For reasons not yet fully elucidated the same pathogenic mutation can lead to a variety of symptoms and the same clinical symptoms can be seen with multiple different mutations. The clinical syndromes associated with the mutations in the present cohort are given in ESI Table 1.† The pathogenic m.3243A > G variant is the most commonly detected heteroplasmic, mitochondrial DNA (mtDNA) variant.^23^ The majority of pathogenic mtDNA variants are heteroplasmic, that is, there is co-existence of both wild type and mutated mtDNA within the same cell or tissue. Variant heteroplasmy levels (expressed as a percentage) are shown in ESI Table 1.† Single, large-scale mtDNA deletions are also heteroplasmic and lead to the loss of both mitochondrial messenger RNA (mt-mRNA) and mitochondrial transfer RNA (mt-tRNA) genes.^24^ POLG-related mitochondrial disease cases harbour recessively-inherited variants in the *POLG* gene encoding the catalytic subunit of mitochondrial polymerase gamma (pol γ), which is required for the replication of mitochondrial DNA.^25^

### Raman spectroscopy

The fibre optic Raman probe has been described previously^26^ (see ESI Fig. 1†). Briefly, a 0.5 mm fibre-optic Raman probe was housed within a standard 21-guage hypodermic needle and coupled to a 830 nm semiconductor laser (Innovative Photonics Solutions); laser power was 60 mW at the probe tip. The probe consisted of a single, low OH laser delivery fibre and an identical Raman collection fibre. An in-line laser wavelength bandpass filter (Semrock Inc USA.) positioned ∼15 cm from the distal tip was used to remove inelastically scattered light produced by Raman scattering and fluorescence generated within the laser delivery fibre. A similar long pass filter (Semrock Inc. USA) was used to prevent elastically scattered light from generating spurious Raman and fluorescence signals in the return fibre. (Thorlabs, Inc.). The probe provided a recording volume of ∼0.25 mm^3^. The collecting fibre was optically coupled to the spectrometer (Raman Explorer Spectrograph, Headwall Photonics, Inc. and iDus 420BR-DD CCD camera, Andor Technology, Ltd).

Samples were stored at −80 °C and thawed to room temperature for measurements. The Raman signal was recorded through a 40 seconds exposure. Spectra were collected from 2–6 sites, depending on the size of the sample.

### Analysis

Analysis was done in MATLAB (R2021b). Using custom scripts, spectra from each sample were averaged (thus, one spectrum equates to one sample in the analysis).^27^ The coefficients of variation for each sample (*i.e.*, the intra-patient variability) at each wavenumber are shown in ESI Fig. 2.† Averaged spectra were interpolated and windowed between 900 cm^−1^ and 1800 cm^−1^; at <900 cm^−1^ spectra were dominated by silica-related background in the optical fibres, at >1800 cm^−1^ spectra consisted of uninformative noise. Spectra were then smoothed (2nd order Savitzky–Golay filter, 5 data point window width) and spectral background was removed using the adaptive, iteratively reweighted penalized least squares (airPLS) algorithm.^28^ Data were then normalised (standard normal variate normalisation). For multivariate analyses, data were mean centred prior to principal component analysis (PCA). Prior to linear discriminant analysis (PCA-LDA), *t*-tests were undertaken and only those PCs manifesting significant between group differences were fed into the LDA. For partial least squares discriminant analysis (PLS-DA) a maximum of 10 latent variables were used and the number of latent variables increased until model performance no longer improved. Classification performance was assessed through leave-one-sample-out cross validation. Accuracy, sensitivity, specificity and area under the receiver operating characteristic curve (AUROC) were computed.

## Results and discussion

### Muscle disease *vs.* no muscle disease

**Fig. 1:**
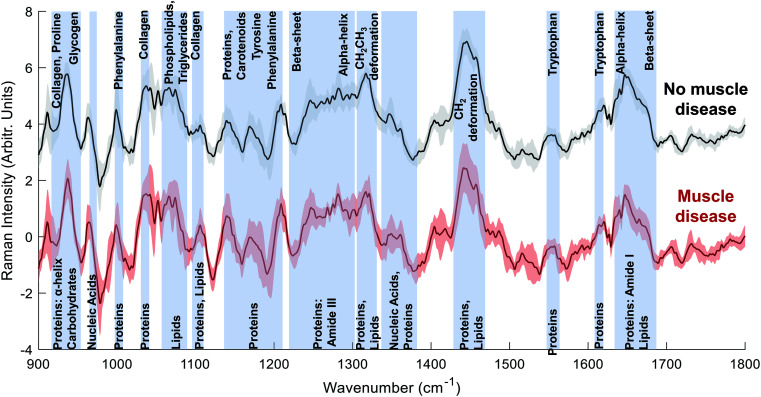
Raman spectra: muscle biopsy samples from patients diagnosed as having or not having muscle disease. Mean ± (s.d) spectra from patients with no pathological evidence of muscle disease (black, top) and those with muscle disease (red, bottom). Key spectral regions are annotated with blue bars.

The Raman spectra of both the muscle disease and no muscle disease groups comprised peaks characteristic of muscle tissue such as amide III (1250–1305 cm^−1^), the CH_2_ deformation of proteins/lipids (1446 cm^−1^) and amide I (1647 cm^−1^) (Fig. 1). Tentative peak assignments were drawn from the available literature.^29–32^ Difference spectra demonstrated that samples from patients without muscle disease had a relative preservation of 930 cm^−1^ (proline), 1080 cm^−1^ (phospholipids), 1123 cm^−1^ (proteins/lipids), 1337 cm^−1^ (nucleic acids), 1451 cm^−1^ (protein/lipids) and 1655 cm^−1^ (amide I).

**Fig. 2:**
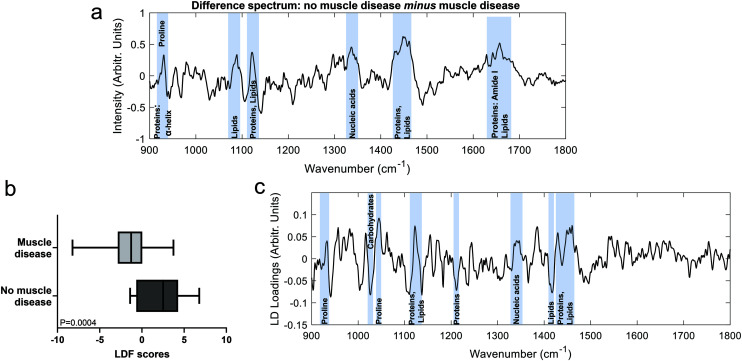
Comparison of muscle biopsy samples from patients diagnosed as having or not having muscle disease. (a). Difference between means spectrum (no muscle disease minus muscle disease) with prominent peaks highlighted. (b). LDF scores from the PCA-LDA analysis. (c). The linear discriminant with prominent spectral regions highlighted.

To further investigate differences between the groups, multivariate analyses (PCA-LDA) were performed (Fig. 2). Significant differences in the linear discrimination function scores were observed between the two groups (Fig. 2c). The distribution of linear discriminant function scores is shown in ESI Fig. 3,† both for the averaged spectra (one spectrum per sample) and for unaveraged spectra (*i.e.* multiple spectra per sample). The linear discriminant loadings plot demonstrated positive contributions at 935 cm^−1^ (protein/glycogen), 1045 cm^−1^ (proline), 1123 cm^−1^ (proteins/lipids) 1340 cm^−1^ (nucleic acids) and 1450–60 cm^−1^ (proteins/lipids). Negative contributions were seen at, for example, 1025 cm^−1^ (carbohydrates) and 1420 cm^−1^ (lipids). Classification accuracy was 70.5% for PCA-LDA and 77.3% for PLS-DA (Table 2).

**Table 2:** Classification performance of fibre optic Raman spectroscopy in the identification of muscle disease

	AUROC	Accuracy	Sensitivity	Specificity
PCA-LDA	0.74	70.5%	58.8%	77.8%
PLS-DA	0.87	77.3%	76.5%	77.8%

In these analyses’, spectra were collected prior to any sample preparation and without targeting the light to discrete areas of the sample manifesting disease related changes, as is sometimes done in work examining the biomedical utility of Raman spectroscopy (*e.g.* ref. 31 and 33). Despite this untargeted approach to spectral acquisition, peaks from both the difference and multivariate analyses suggest an overall reduction in α-helical protein content (*e.g.* peaks at 935, 1300, 1655 cm^−1^) in disease, which we have previously demonstrated in a mouse model of myopathy.^29^ Similar changes have also been reported in fly models of myopathies^20^ and might therefore represent a translational biomarker of muscle disease.

### Different muscle diseases: mitochondrial myopathy *vs.* non-mitochondrial myopathy

**Fig. 3:**
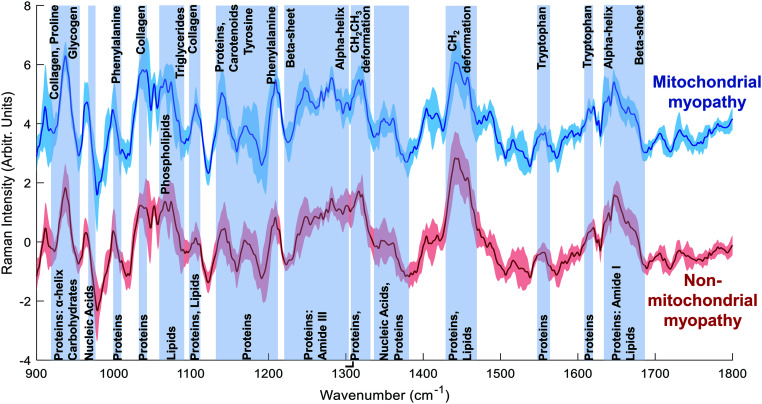
Raman spectra: mitochondrial myopathy and non-mitochondrial myopathy groups. Mean ± (s.d) spectra from patients with mitochondrial disease (blue, top) and those with non-mitochondrial myopathy (red, bottom).

**Fig. 4:**
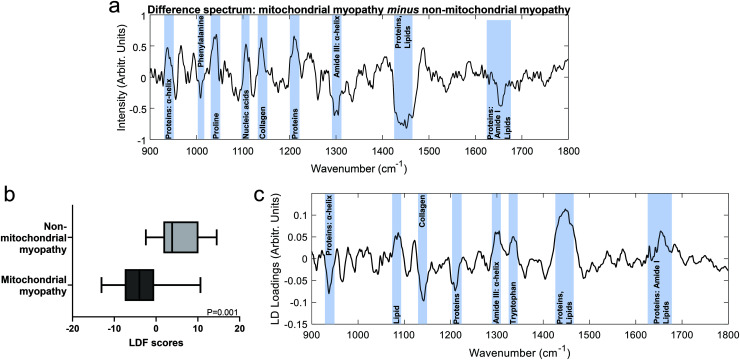
Comparison of mitochondrial myopathy and non-mitochondrial myopathy groups. (a). Difference between means spectrum (mitochondrial myopathy minus non-mitochondrial myopathy) with prominent peaks highlighted. (b). LDF scores from the PCA-LDA analysis. (c). The linear discriminant prominent spectral regions highlighted.

Mean spectra for mitochondrial myopathy and non-mitochondrial myopathy are shown in Fig. 3. Difference spectra between patients with mitochondrial myopathy and those with other causes of myopathy (“non-mitochondrial myopathy”) demonstrated that peaks at 1040 cm^−1^ (proline), 1105 cm^−1^ (carbohydrates), 1140 cm^−1^ (collagen) and 1210 cm^−1^ (proteins) were more prominent in mitochondrial myopathy (Fig. 4). Peaks at 1005 cm^−1^ (phenylalanine), 1300 cm^−1^ (amide III: α-helix, lipids) cm^−1^, 1451 cm^−1^ (proteins/lipids) and 1655 cm^−1^ (amide I, α-helix) were seen in non-mitochondrial muscle disease samples (Fig. 4). These could suggest a relative preservation of α-helical protein content in non-mitochondrial myopathy samples.^34,35^ In the LDF plots, positive contributions were seen at 1087 cm^−1^ (lipid), 1300 cm^−1^ (amide III, α-helix), 1334 cm^−1^ (tryptophan), 1451 cm^−1^ (proteins/lipids) and 1655 cm^−1^ (amide I). Negative contributions were seen at 935 cm^−1^ (protein/glycogen), 1140 cm^−1^ (collagen) and 1210 cm^−1^ (protein). The distribution of LDF scores is shown in ESI Fig. 4.† Classification accuracy (using averaged spectra) was 74.1% for PCA-LDA and 88.9% for PLS-DA (Table 3).

**Table 3:** Classification performance of fibre optic Raman spectroscopy in the identification of mitochondrial myopathy and non-mitochondrial myopathy

	AUROC	Accuracy	Sensitivity	Specificity
PCA-LDA	0.76	77.8%	78.6%	76.9%
PLS-DA	0.95	88.9%	85.7%	92.3%

The small number of patient samples available for study places limitations on the interpretation of the classification performance of these data. Notwithstanding the challenges of collecting muscle samples from patients with relatively rare conditions, validation of the present results through a larger study would help further explore the utility of Raman spectroscopy for the diagnosis of muscle disorders. Such a study would also permit testing of additional and more sophisticated machine learning algorithms, which may improve diagnostic performance.^36^ A larger trial would also be able to explore if Raman spectra can be used to further classify specimens, for example, into different genetic subtypes of mitochondrial myopathy (as well as mitochondrial disease without myopathy), or into dystrophic or inflammatory myopathies. If these distinctions were possible, Raman analyses could potentially help direct further tests such as immunohistochemical and/or genetic analyses. An extensive mapping of samples may also be useful in determining the spectral fingerprint associated with discrete pathological features such as atrophied and regenerating muscle fibres, inflammatory cell infiltrates and connective tissue changes. It is presently unclear how well data collected on high resolution mapping microscope formats will transfer to fibre optic systems, but significant advances are being made in the transference of data from one system to another.^37,38^

One of the attractions of fibre optic Raman spectroscopy is the possibility for *in vivo* measurements. Using the same equipment, we have recently described *in vivo* intra-muscular recordings in mouse models of two neurological diseases (amyotrophic lateral sclerosis and Duchenne muscular dystrophy).^29^ As those recordings did not appear to have any deleterious effects upon living muscle, similar recordings in human patients may also be possible. Any implementation of *in vivo* intra-muscular Raman spectroscopy will require approval from relevant regulatory authorities, such as the Medicines and Healthcare products Regulatory Agency (MHRA) in the UK. For *in vivo* clinical translation, the equipment used herein would require some adaptations, such as sterilised and disposable fibre optics within the standard hypodermic needle. Minimising the length of the optical fibres would also limit unwanted background signal. Despite these potential issues, significant progress has been made in testing fibre optic Raman technologies in human subjects *in vivo.*^39^ The translation of Raman spectroscopy into clinical use also faces several challenges around the complex data analysis, data sharing and equipment. Initiatives such as the UK EPSRC Clinical Infrared and Raman Spectroscopy Network and the EU COST Action Raman4Clinics are progressing the development of standard operating procedures and raising awareness of the potential clinical utility of vibrational spectroscopy.^40^

## Conclusions

In this study we have explored the potential of spontaneous fibre optic Raman spectroscopy to identify muscle disease in human samples prior to any sample preparation. Our data demonstrated a high classification accuracy for both the identification of muscle disease, as well as differentiation of two different classes of muscle disease. As the fibre optic probe can be sited within a standard hypodermic needle,^26^*in vivo* measurements may be achievable and could augment the existing diagnostic tools, with the potential for real-time, bedside diagnostics. Thus, this work represents an important early step in the application of Raman spectroscopy to muscle diseases.

## Author contributions

JJPA: conceptualisation, data curation, formal analysis, funding acquisition, investigation, methodology, project administration, resources, visualisation, writing – original data; MP: investigation, project administration, writing–review & editing; GRL: software, writing–review & editing; APD: investigation, writing–review & editing; CAK: funding acquisition, investigation, writing–review & editing; CH: resources, methodology, writing–review & editing, MH – resources, writing–review & editing; CJM: conceptualisation, resources, writing–review & editing, GSG: conceptualisation, resources, writing–review & editing; RWT: conceptualisation, resources, writing–review & editing; PJS: conceptualisation, funding acquisition, resources, writing–review & editing; JCCD: conceptualisation, funding acquisition, investigation, methodology, project administration, resources, writing–review & editing.

## Conflicts of interest

There are no conflicts to declare.

## Supplementary Material

AN-147-D1AN01932E-s001

AN-147-D1AN01932E-s002
