# Supplementary material for: Rapid identification of human muscle disease with fibre optic Raman spectroscopy
Source: Analyst. 2022 Apr 21;147(11):2533–40. doi: 10.1039/d1an01932e (PMC9150427; doi:10.1039/d1an01932e)
Supplement: AN-147-D1AN01932E-s001 [file AN-147-D1AN01932E-s001.pdf]

Electronic Supplementary Material (ESI) for Analyst.
This journal is © The Royal Society of Chemistry 2022

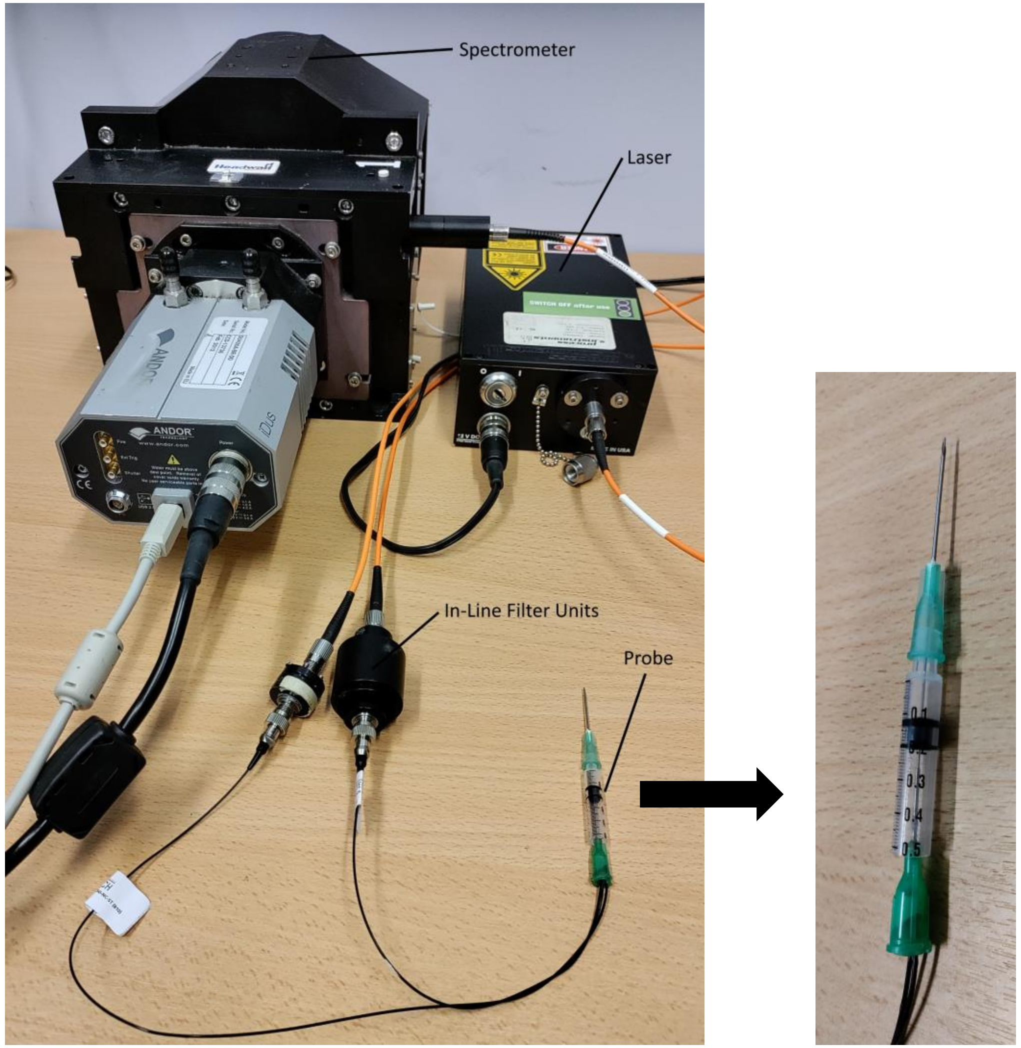

Supplemental figure 1. The fibre optic Raman system.

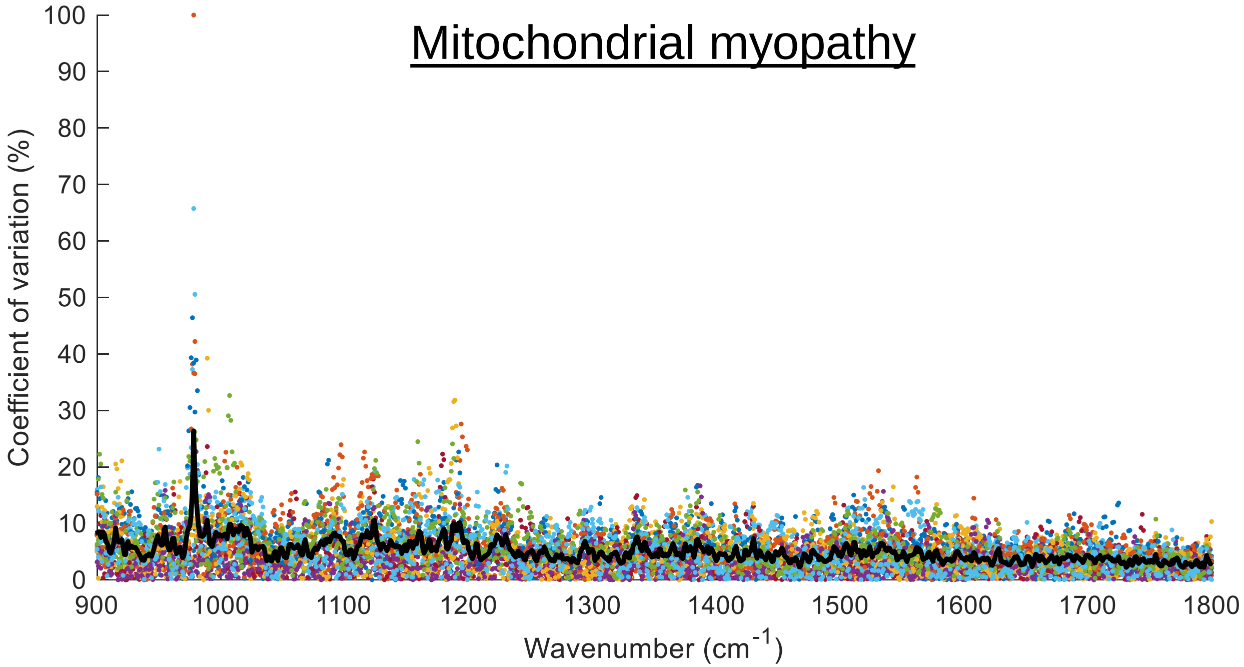

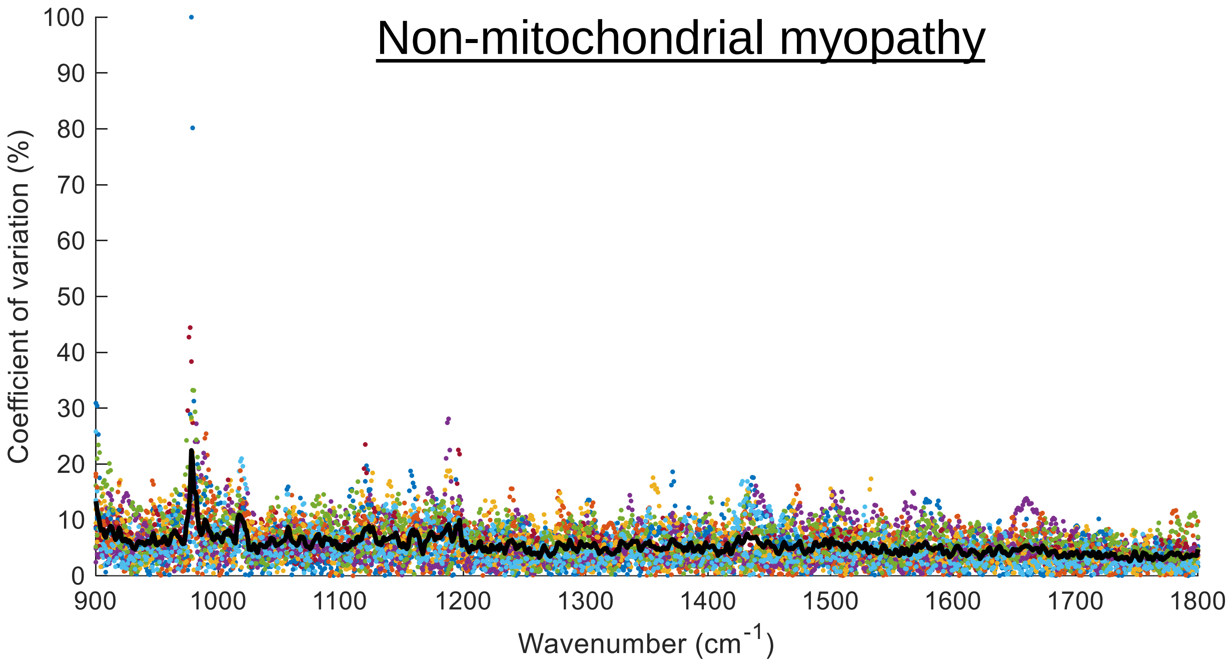

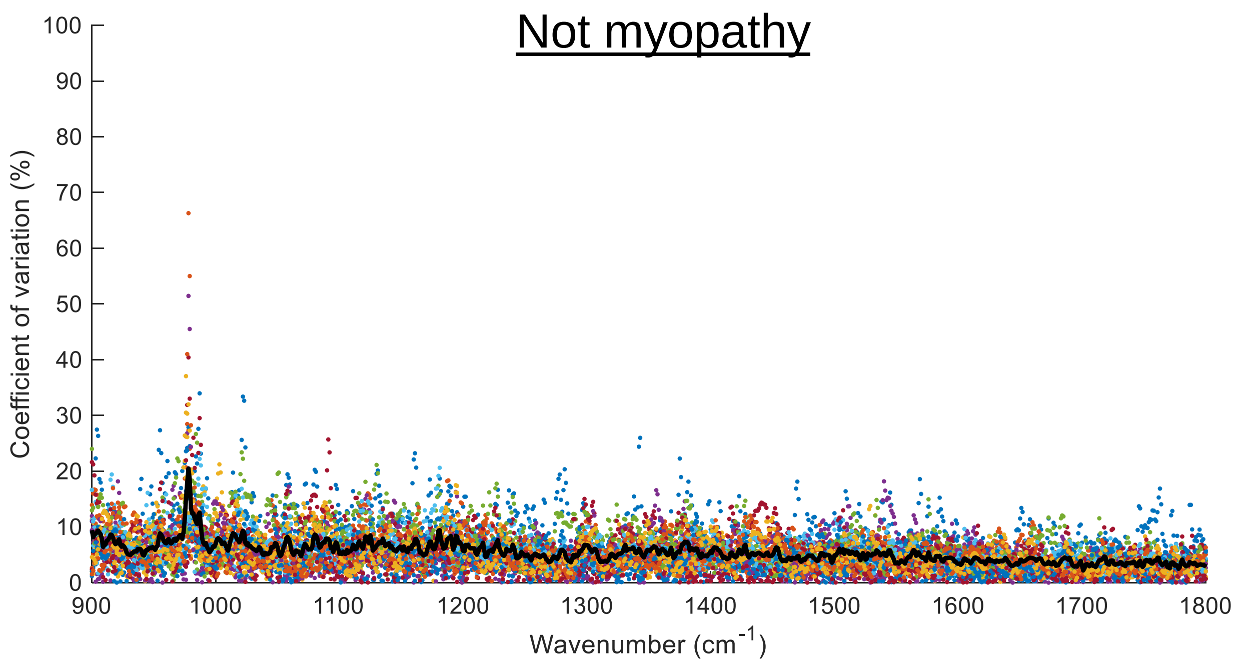

Supplemental figure 2. Intra-patient variability.

The variation of spectra within each sample was captured through calculation of the coefficient of variation at each wavenumber for each sample. The dots are the individual values at each wavenumber for every spectrum, coloured by sample. The average at each wavenumber is shown as a black line.

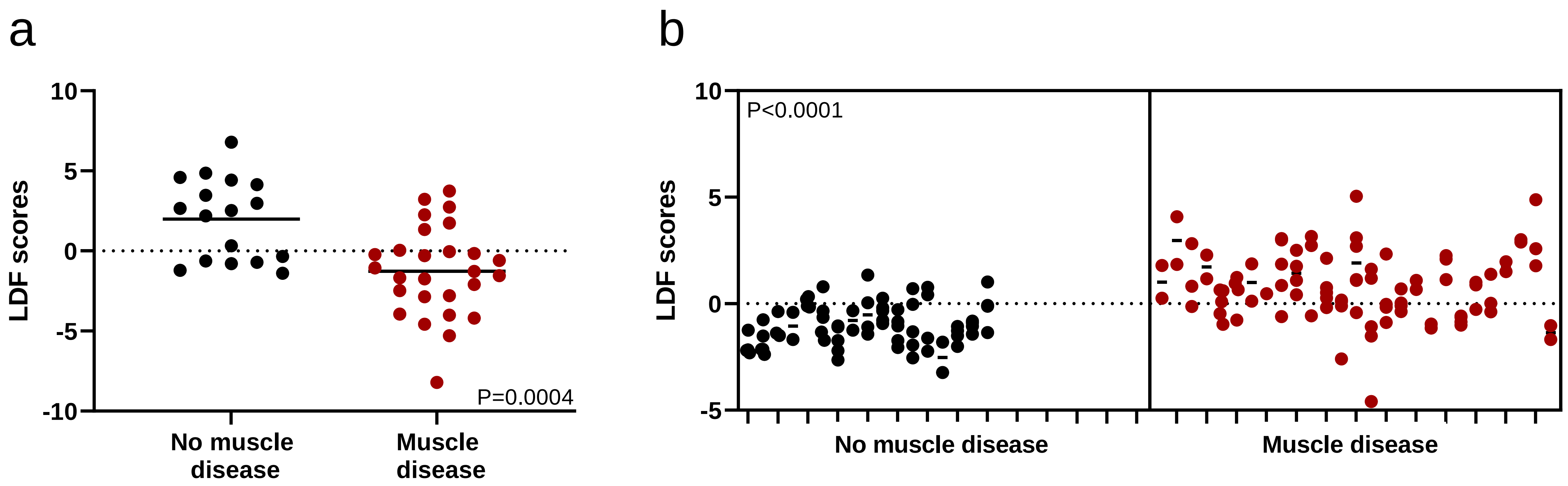

Supplemental figure 3. LDF scores of averaged spectra and unaveraged spectra: no muscle disease vs. muscle disease.

a). In the main text spectra from individual muscle samples were averaged prior to PCA-LDA analysis. A scatter plot of the LDF scores obtain via this approach is shown.

b). Unaveraged spectra can also be fed into the PCA-LDA analysis. 40 PCs were investigated (>90% of the variance of the data). Using the same algorithm, 4 PCs were chosen as inputs to the LDF and a plot of nested LDF scores (each spectrum for each sample) is shown. P value shown is from a nested t-test. Note that the LDF is now using a different feature space and the positive/negative LDF scores in the two plots are not directly comparable.

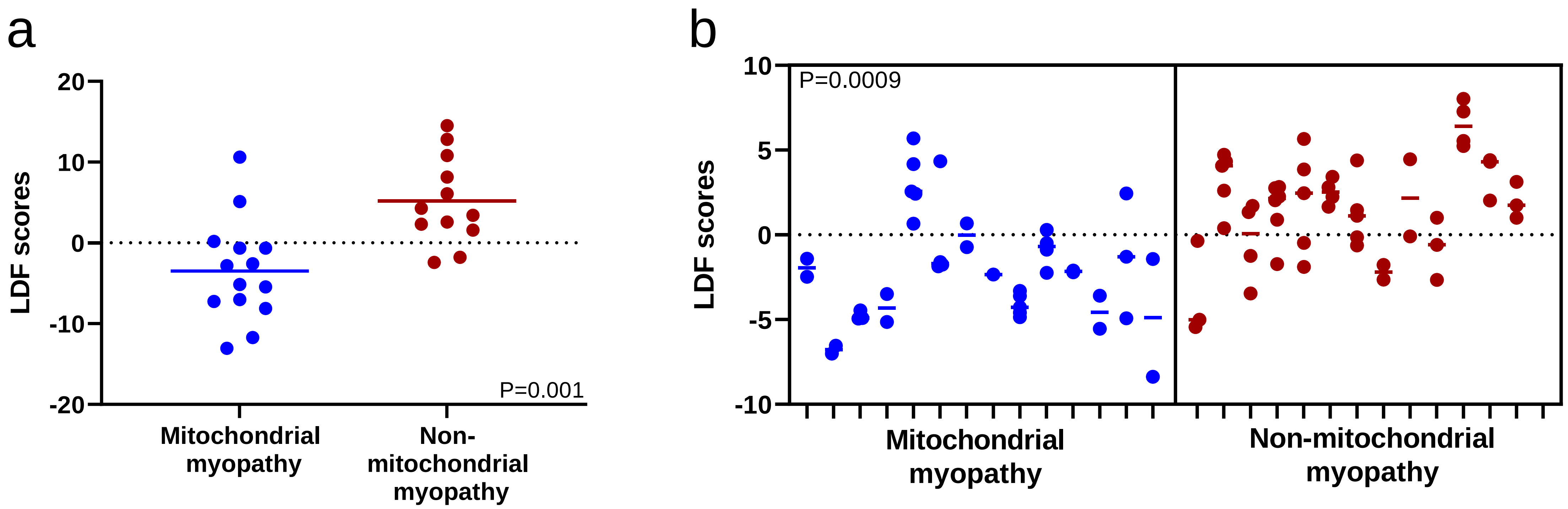

Supplemental figure 4. LDF scores of averaged spectra and unaveraged spectra: non-mitochondrial myopathy vs. mitochondrial myopathy.

a). In the main text spectra from individual muscle samples were averaged prior to PCA-LDA analysis. A scatter plot of the LDF scores obtain via this approach is shown.

b). Unaveraged spectra can also be fed into the PCA-LDA analysis. 40 PCs were investigated (covering >90% of the variance of the data). Using the same algorithm as that detailed in the methods of the main text, 2 PCs were chosen as inputs to the LDF and a plot of nested LDF scores (each spectrum for each sample) is shown. P value shown is from a nested t-test.
