# Supplementary material for: Rapid identification of human muscle disease with fibre optic Raman spectroscopy
Source: Analyst. 2022 Apr 21;147(11):2533–40. doi: 10.1039/d1an01932e (PMC9150427; doi:10.1039/d1an01932e)
Supplement: AN-147-D1AN01932E-s002 [file AN-147-D1AN01932E-s002.pdf]

Electronic Supplementary Material (ESI) for Analyst.
This journal is © The Royal Society of Chemistry 2022

MITOCHONDRIAL DISEASE						
**Gender**	**Age**	**Clinical phenotype**	**Muscle**	**Biopsy findings**	**Genotype**	**Muscle heteroplasmy (%)**
F	62y	Maternally inherited diabetes and deafness	TA	3% COX-deficient fibres, 1% ragged-red fibres	m.3243A>G	38
F	29y	Mitochondrial encephalopathy, lactic acidosis, and stroke-like episodes	VL	<1% COX-deficient fibres	m.3243A>G	50
M	31y	Asymptomatic	VL	4% COX-deficient fibres, 2% ragged-red fibres	m.3243A>G	63
F	53y	Maternally inherited diabetes and deafness	TA	4% COX-deficient fibres, 2% ragged-red fibres	m.3243A>G	73
F	55y	Maternally inherited diabetes and deafness, Left ventricular hypertrophy, GI dysmotility, Mild ataxia	TA	1% COX-deficient fibres	m.3243A>G	84
M	49y	Maternally inherited diabetes and deafness	TA	4% COX-deficient fibres, 2% ragged-red fibres	m.3243A>G	74
M	44y	Subtle progressive external ophthalmoplegia, myopathy, exercise intolerance SNHL Glucose intolerance	VL	% COX-deficient fibres	m.3243A>G	68
M	29y	Progressive external ophthalmoplegia, ptosis, exercise intolerance	TA	40% COX-deficient fibres, 20% ragged-red fibres	Single, large-scale mtDNA deletion	Not determined

F	45y	Maternally inherited diabetes and deafness	TA	1% COX-deficient fibres	m.3243A>G	37
M	54y	Mitochondrial encephalopathy, lactic acidosis, and stroke-like episodes	TA	25% COX-deficient fibres, 10% ragged-red fibres	m.3243A>G	64
M	58y	Myoclonic epilepsy with ragged red fibres. Patient 6 in[1]	TA	30% COX-deficient fibres, 8% ragged-red fibres	m.8344A>G	90
M	80y	Progressive external ophthalmoplegia, ptosis, generalised myopathy. Patient 10 in[2]	TA	20% COX-deficient fibres, 5% ragged-red fibres	Recessive *POLG* variants (p.Ala467Thr; p.Thr251Ile/p.Pro587Leu)	N/A
M	56y	Progressive external ophthalmoplegia, ptosis, peripheral neuropathy, epilepsy. Patient 5 in[3]	TA	17% COX-deficient fibres, 8% ragged-red fibres	Recessive *POLG* variants (p.Trp748Ser; p.Arg1096Cys)	N/A
F	64y	Ptosis, peripheral neuropathy, ataxia	Delt	15% COX-deficient fibres, 4% ragged-red fibres	Recessive *POLG* variants c.2542G>A p.(Gly848Ser); c.2799T>G p.(Ser933Arg)	N/A
**NON-MITOCHONDRIAL MYOPATHY**						
**Gender**	**Age**	**Clinical phenotype**	**Muscle**	**Main biopsy findings**	**Other investigation results**	**Clinico-pathological diagnosis**
M	45y	Proximal upper and lower limb weakness. Chronic renal failure (known cause), on dialysis	Biceps	Type II fibre atrophy, few moth eaten fibres on NADH staining	-	Metabolic (uraemic) myopathy

F	69y	Proximal upper limb weakness	Deltoid	Excess of type I fibres, few moth eaten fibres on NADH staining. Fat deposition between muscle fibres. <1% COX deficient fibres	CK 87	Myopathy: unknown aetiology
F	61y	Proximal muscle weakness, fatigue, muscle pain	Deltoid	Excess of type I fibres	CK 62, weak anti-nuclear antibody positive. Extractable nuclear antigen and myositis antibody negative	Myopathy: unknown aetiology
M	73y	Proximal upper and lower limb weakness	Quads	Some central loss of NADH staining, occasional course architecture to fibres.	CK 62 Sensorimotor axonal neuropathy	Myopathy: unknown aetiology
M	59y	Lower limb weakness, muscle hypertrophy	Quads	Scattered atrophic fibres, mainly type II. Excess nuclear internalisation. No lipid or glycogen. Necrotic fibres seen win association with macrophages. MHC class I upregulation. No inflammatory cells.	Anti-voltage gated potassium channel positive.<br><br>CASPR-2, LGI-1 negative.<br><br>Extractable nuclear antigen and myositis antibody negative	Subacute idiopathic inflammatory myopathy<br><br>(Immune mediated necrotising myopathy)
M	39y	Proximal leg weakness	Quads	Type I fibre predominance, variation in fibre size. Excess nuclear internalisation. Marked inflammation, presumed to be secondary to dystrophic process. Marked fibre splitting. Connective tissue expansion and fatty replacement	CK 284. FHL1 gene negative	Dystrophic myopathy<br><br>(Unclassified limb girdle muscular dystrophy)

				of myocytes.		
M	56y	Proximal lower limb weakness, muscle pain.	Quads	Lymphocytic infiltration. MHC-1 upregulation. COX negative fibres.		Inclusion body myositis
M	56y	Leg weakness, then arm weakness. Dysarthria, dysphagia	Deltoid	Scattered atrophic fibres, some fibre splitting and fragmentation. Excessive nuclear internalisation. Moth-eaten fibres on MADH staining. Normal IHC for dystrophins 1-3, sarcoglycans, emerin, dysferlin and caveolin-3.	CK 444	Dystrophic myopathy
M	64y	Proximal leg weakness	Deltoid	Slight excess of nuclear internalisation. No lipid or glycogen accumulation. No necrosis. Subsarcolemmal accentuation on NADH and trichrome stains. Subsarcolemmal accumulations of mitochondria on EM, no COX negative fibres, no red ragged fibres.	CK 63	Myopathy: unknown aetiology
M	22y	Proximal weakness, facial weakness, long finger flexor and elbow contractures	Quads	Significant variation in fibre size. Necrotic fibres seen. Increased internal nuclei. Fibre splitting and	CK 901. LMNA, VCP, MYH7, collagen 6 and 12, STIM1, ORA1, FSHD1 and 2 all negative.	Dystrophic myopathy<br><br>(Limb girdle muscular dystrophy)

				hypertrophy. Lipid and glycogen normal. Dysferrlin reduced.	100,000 genome negative	
F	25y	Proximal upper and lower limb weakness	Quads	Non-specific myopathic changes. Fibre size variation, excess nuclear internalisation and slight excess endomysial collagen	Positive anti-SRP	Subacute idiopathic inflammatory myopathy<br><br>(Anti-SRP related myopathy)
F	35y	Hand grip weakness and proximal lower limb weakness	Biceps	Patchy core-like areas of lost NADH reactivity.	CK 91. Heterozygous change in TTN gene (c.7304T>G, p.(Tyr24349	Dystrophic myopathy<br><br>(TTN myopathy)
M	72y	Longstanding upper limb weakness, late onset lower limb weakness	Deltoid	Increased variation in fibre size - atrophic and hypertrophic. Increased nuclear internalisation. No excess lipid of glycogen. Chronic myopathic process	CK 217	Myopathy: unknown aetiology
**NO MUSCLE DISEASE**						
**Gender**	**Age**	**Clinical phenotype**	**Muscle**	**Main biopsy findings**	**Other investing. results**	**Clinico-pathological diagnosis**
F	61	Cognitive decline, deaf.	Quads	<2% COX negative fibres. Age-related changes.	Subcortical WM changes on MRI	Vascular dementia
F	80y	Ataxia, myoclonus	Deltoid	Some type II fibre atrophy. 2% COX deficient fibres, <1% red ragged fibres. Likely age-related changes. Some evidence of low-level	No evidence of m.324a>G, MTTL1 pathogenic variant, no pathogenic mitochondrial DNA mutation	Ataxia of uncertain cause

				mitochondrial DNA re-arrangements, felt unlikely to be responsible for symptoms.	associated with NARP/MILS, no mitochondrial DNA variance associated with the MERRF phenotype.	
F	59y	Occasional dysphagia & ptosis, leg pain	Quads	Very few atrophic fibres (of both fibre types), otherwise normal.	CK 113. Anti-AChR antibody negative.	Levator palpebrae dehiscence, L4/5 spinal canal stenosis
M	77y	Proximal weakness	Deltoid	Very few red ragged and COX negative fibres, thought to be age-related.	CK 97. Anti-AChR antibody positive	Myaesthenia gravis + osteoarthritis
M	23y	Myalgia	Quads	Normal	CK 362	Fibromyalgia
F	42y	Falls, ataxia	Deltoid	Normal	Anti-GAD negative. ANA and ENA negative. Coeliac screen negative	Ataxia of uncertain cause
M	33y	Renal angle pain one week following general anaesthetic.	Biceps	Normal	CK 23,000	Malignant hyperthermia
F	40y	Sensory ataxia comprising patchy numbness and loss of balance.	Deltoid	Normal	CK 43. Anti-TTG antibodies positive+. Nerve conduction studies – asymmetric, non-length dependent sensory nerve abnormalities	Coeliac disease-related sensory ganglionopathy

M	64y	Incidentally raised CK	Quads	Normal	CK 800. Myositis antibodies and HMGCo-A reductase negative.	Statin related hyper CK-aemia
F	74	Late onset cerebellar ataxia, deafness and myoclonus. Falls	Deltoid	Few atrophic type II fibres, <1% cox negative. No lipid/glycogen accumulation. No mitochondrial DNA abnormalities	Positive IgG anti-TG6 antibodies	Gluten related cerebellar ataxia
M	71	Ataxia and dysarthria	Deltoid	Atrophic type II fibres. 2% COX negative fibres. Rare red ragged fibres. Non-specific mitochondrial appearances.	Positive IgG anti-TG6 antibodies	Gluten-related cerebellar ataxia
F	61y	Ataxia, nystagmus	Deltoid	Increased variation in fibre size due to atrophic type II fibres. No group atrophy. Normal mosaic pattern of fibre type distribution. Sparse COX negative fibres (0.1%).	ANA, coeliac, monoclones, melas/merrf/narp genetics all negative. Ataxia genetic panel negative	Ataxia, uncertain aetiology
M	77y	Ataxia	Deltoid	Sparse COX negative fibres (<0.5%). Otherwise normal	Ataxia genetics negative	Ataxia of uncertain aetiology
F	59y	Ataxia, epilepsy, deafness	Biceps	Sparse COX negative fibres (<1%). Otherwise normal. Mitochondrial DNA sequencing normal.	Ataxia genetics negative	Ataxia of uncertain aetiology

F	37y	Learning difficulties, deaf, epilepsy, ataxia	Deltoid	Areas of staining loss with both NADHTR and COX/SDH preparations; considered non-specific finding. Normal EM.	CK 117 Ataxia genetic panel negative. SCA 1/2/3/6/7 and Friedrich's ataxia genetics negative	Likely genetic disorder, no cause found
F	54y	Lower limb weakness and pain. 3rd nerve palsy.	Biceps	Mild type II atrophy only, otherwise normal	Sensorimotor neuropathy	Diabetes related neuropathy and 3rd nerve palsy
F	31y	Leg spasms, back pain, nausea. Hiatus hernia, gastrotomy and gastric dumping syndrome	Biceps	MHC1 upregulation of sarcolemmal membrane staining. Very occasional perivascular aggregates of inflammatory cells. No morphological evidence of myopathy.	CK 75. HSP genetics negative	Repeated IM injections of cyclizine for refractory nausea causing inflammation and fibrosis.

Supplementary table 1. Clinico-pathological characteristics of the patients.

M – male, F – female, Y – years, COX – cytochrome C oxidase, CK – creatine kinase, NADH - Nicotinamide adenine dinucleotide-hydrogen (reduced form of NADH), Quads – quadriceps muscle, CASPR-2 – Contactin associated protein red-2, LGI-1 - Leucine-rich glioma-inactivated 1 antibody, MERRF – Myoclonic epilepsy with ragged red fibres; MHC1 – major histocompatibility complex-1; MILS - Maternally Inherited Leigh Syndrome ; MTTL1 - Mitochondrially encoded tRNA leucine 1; NARP - neurogenic muscle weakness, ataxia, and retinitis pigmentosa; SRP – signal recognition particle; AChR – acetylcholine receptor; TTG – tissue transglutaminase; HMGCo-A - β-Hydroxy β-methylglutaryl-CoA; SCA – spinocerebellar ataxia; HSP – hereditary spastic paraparesis.
